# Xeno-monitoring the impact of Vector Control on trypanosome transmission in the Forecariah sleeping sickness focus (Guinea)

**DOI:** 10.1371/journal.pntd.0013598

**Published:** 2026-06-05

**Authors:** Abdoulaye Dansy Camara, Patrice Boedouno, Issiaga Camara, Aissata Soumah, Mohamed Kébé, Jeannette Koivogui, Brice Rotureau, Jean-Mathieu Bart, Bruno Bucheton, Alexandre Delamou, Adrien Marie Gaston Belem, Moise Kagbadouno, Mamadou Camara

**Affiliations:** 1 National Control Program for Neglected Tropical Diseases, Conakry, Guinea; 2 National Center for Training and Research in Rural Health of Maferinyah, Forecariah, Guinea; 3 Parasitology Unit, Institut Pasteur of Guinea, Conakry, Guinea; 4 Intertryp, Univ Montpellier, CIRAD, IRD, Montpellier, France; 5 Gamal Abdel Nasser University Conakry, Conakry, Guinea; 6 Nazi Boni University, Bobo-Dioulasso, Burkina Faso; University of Illinois at Urbana-Champaign, UNITED STATES OF AMERICA

## Abstract

**Background:**

Forecariah is one of the most active foci of *gambiense* human African trypanosomiasis (gHAT) in Guinea. The study aimed to evaluate the impact of a vector control intervention carried out in this focus from 2018 to 2021. To achieve this, thousands of deltamethrin-impregnated blue tiny targets were deployed annually.

**Methodology and principal findings:**

Thirty-one sentinel traps were used to assess the following entomological and parasitological parameters: (i) the apparent density (AD) of tsetse flies, monitored twice per year; (ii) the tsetse fly infection rates, evaluated once per year; and (iii) the trypanosome species, determined by molecular approaches before and after three years of vector control. Prior to the VC campaign, significantly higher densities of *Glossina palpalis gambiensis* were observed in the mangroves (AD = 17.2 F/T/D) than in mainland areas (AD = 6.2 F/T/D). Just 18 months after the start of the VC campaign, the maximum reduction in AD was reached (mean AD = 1.1 F/T/D; p < 0.001; 92% reduction) and was maintained throughout the campaign. Prior to VC, 18 out of 31 (58%) sentinel traps presented at least one infected fly observed by microscopy, whereas only two out of 31 (6%; p = 0.002) traps were positive after three years of VC. Molecular analysis of the tsetse midgut revealed positivity for *T. brucei* sl DNA in 45.2% of the traps prior to the VC baseline. *T. congolense* and *T. vivax* were observed in 6.5% of traps. Although one *T. b. gambiense* infection, which is responsible for human disease, was detected three years after the introduction of vector control, a significant decrease in *T. brucei* sl PCR positivity was observed (45.2% versus 22.6%; p = 0.05), whereas a slight increase was observed for *T. vivax* (6.5% versus 16.1%), while *T. congolense* trap positivity (6.5%) remained stable.

**Conclusion:**

In Forecariah, the prevalence of the disease was reduced by 76% during the study period. Hence, these results further emphasize that implementing vector control alongside medical control is an effective way of reducing the transmission of parasites to humans.

## Author summary

In January 2025, the World Health Organization validated the elimination of *gambiense* human African trypanosomiasis (gHAT) as a public health problem in Guinea. This goal was achieved through a combination of medical and entomological activities over one decade. This strategy was successively implemented in the three gHAT active foci, first in Boffa in 2012, then in Dubreka in 2016 and in Forecariah in 2018. Here, we present the evaluation results of the first three years (2018–2021) of vector control (VC) in the Forecariah focus. VC consisted in the annual deployment of over 5,000 blue insecticide-impregnated tiny targets, mainly in mangrove areas, which are at high risk of *Trypanosoma brucei gambiense* transmission through tsetse fly bites. Only 18 months after the initial deployment, the apparent density of tsetse flies (ADT) had decreased by 92%. This ADT drop was associated with a significant reduction of the trypanosome infection rates (from 11.5% to 1.2%) in tsetse midguts over the three years of VC. Prior to VC implementation, at least one infected fly was found in 18 of the 31 sentinel traps, whereas only two were positive after three years of monitoring. A pan-trypanosome PCR, used as a molecular xenomonitoring tool, revealed the predominant presence of *T. brucei* sl., while *T. vivax* and *T. congolense* were detected in few tsetse midguts. During this period, HAT prevalence decreased by 76%, highlighting the effectiveness of this combined strategy.

## Introduction

Transmitted to humans by tsetse, the trypanosome is the causative agent of sleeping sickness or human African trypanosomiasis (HAT), which is still a public health problem in many African countries south of the Sahara and which the WHO aims to eliminate by 2030. Two species are responsible for the disease: *Trypanosoma brucei gambiense* (*T. b. gambiense*) causes the chronic form present in Central and West Africa and *Trypanosoma brucei rhodesiense* (*T. b. rhodesiense*) causes the acute form present in East Africa [1]. The disease occurs in rural areas and causes intermittent fever, cervical lymphadenopathy and sleep disorders. Sleeping sickness can lead to cachexia, drowsiness, coma, or even death in the absence of appropriate treatment. Diagnosis is often late because the clinical symptoms associated with the early diseases stages are poorly specific [2]. The lack of qualified personnel, the absence of chemoprophylaxis and vaccine and the difficulty of diagnosis constitute an obstacle to the elimination of the disease in endemic countries [2,3].

In 2015, the WHO reported that 2801 individuals were infected with this disease [4]. Thanks to the efforts made by the HAT national control programs (HAT-NCP) of endemic countries, a significant reduction has been observed with less than 1000 cases since 2018 [5], thus giving hope for the elimination of the disease. Despite these efforts, the number of cases reported over the past years has remained stable, indicating that transmission persists in some epidemiological settings [6]. One strategy has been advised by the WHO to ensure *gambiense* HAT (g-HAT) elimination: in active transmission areas, screening and treatment of cases to clean up the human reservoir together with targeted vector control in priority areas to lower human/vector contacts [7]. This strategy has been widely used in several countries [8]. The introduction of the tiny targets [9] was an opportunity that has helped to strengthen vector control in g-HAT foci. Studies carried out in this context in Guinea [10], Uganda [11], Democratic Republic of the Congo [12] and Ivory Coast [13], Cameroon [14] showed that the use of vector control along with medical control allowed a significant reduction in the number of cases.

In West Africa, Guinea is the country with the highest number of g-HAT cases today. They are distributed in three active foci delimited by the mangrove areas of Boffa, Dubreka and Forecariah where human populations live in close contact with tsetse [15]. The number of g-HAT cases detected in the 3 foci was 140 and 73 in 2017 and 2018 respectively, including 53 and 30 cases in the Forecariah focus [16].

Despite multiple medical screening campaigns, the level of endemicity has remained stable, with prevalence ranging from 0.5% to 2%, and the rate of population taking part in screening is constantly decreasing [17]. The introduction in 2012 of vector control through the annual deployment of Tiny Targets in the eastern part of the Boffa focus has led to a drastic reduction in prevalence [9]. The Ebola epidemic in 2014 prevented mass screening of the population, leading to an increase in gHAT prevalence in certain areas not covered by the vector control intervention. Interestingly, with the continuation of vector control in Boffa East, no new cases of g-HAT was reported in this area, suggesting that this method could efficiently help to reduce the transmission of *T. b. gambiense* [16].

Based on these strong results, the neglected tropical diseases national control programme (NTD-NCP; previously named HAT-NCP) extended this strategy in 2018 to the Forecariah focus in combination with medical control. During the three years (2018–2021) of this vector control, the NTD-NCP carried out entomological and parasitological evaluations to better understand the impact of this strategy in this focus. Our study was conducted with the general objective of assessing the level of reduction in trypanosome transmission in the vector following the intervention. Specifically, it aimed to determine the density and distribution of the vector and the proportion of infected tsetse, as well as to identify the species of trypanosomes circulating in this focus.

## Materials and methods

### Ethics statement

The protocol for this study was submitted to the Guinean National Ethics Committee for Health Research (CNERS) for approval before the start of any activity (No.: 113/CNERS/22). We also obtained agreement from the authorities of the Program and the Forecariah district for the use of these data.

### Study area

**Fig 1:**
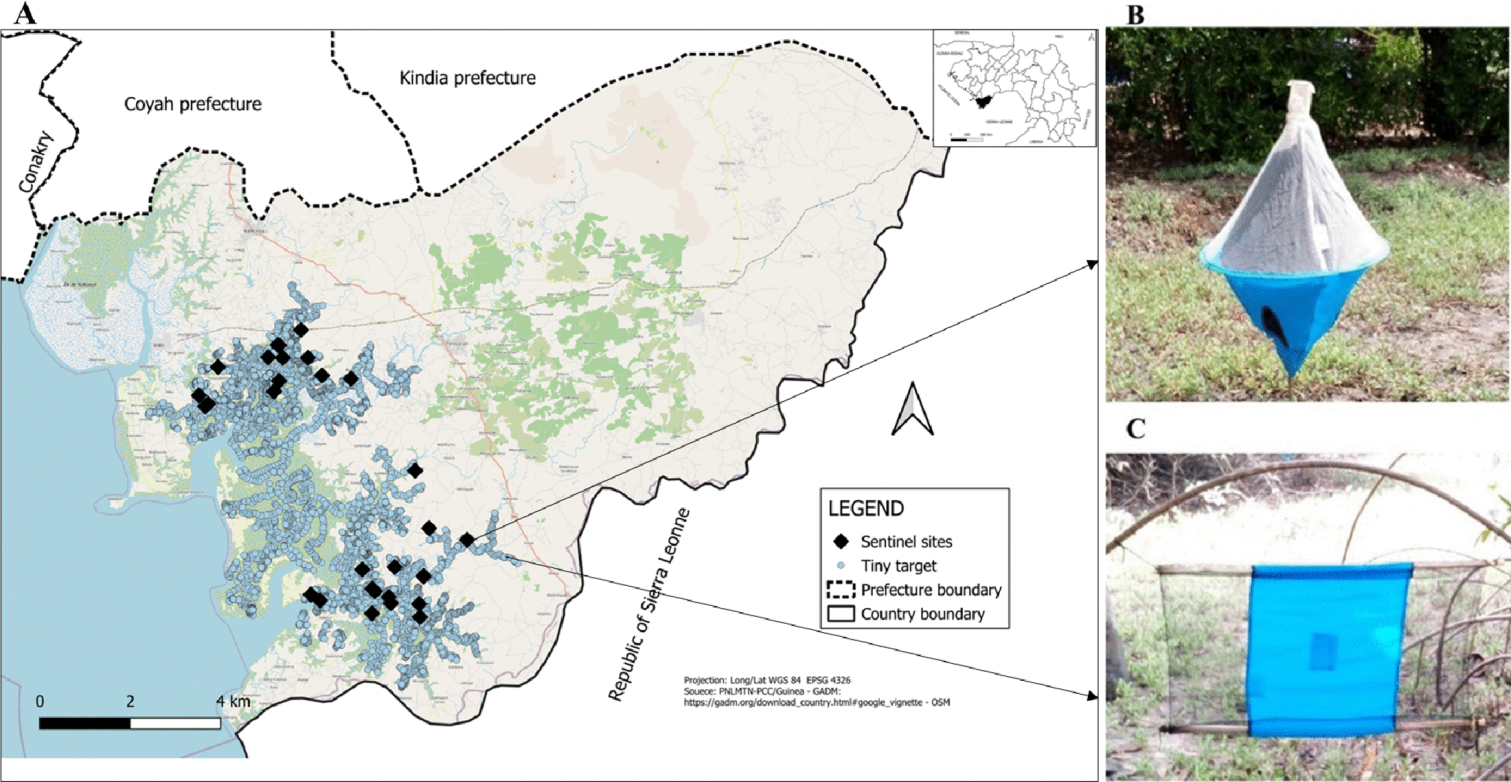
Presentation the Forecariah focus (A); Biconical trap (B); Tiny Target (C). This map was elaborated in-house with QGIS 3.28.12 from an OSM standard layer (www.openstreetmap.org).

The prefecture of Forecariah, one of the three active g-HAT foci in Guinea, is located on the coastal part of the country at the border with the Republic of Sierra Leone (Fig 1A). The landscape of the area has two main aspects. The mainland is characterised by savannah, while the islands are characterised by a mangrove ecosystem. The climate is of humid tropical coastal type with two seasons (dry and rainy) lasting six months each (November - April and May – October respectively). The main human activities are rice cultivation, fishing, salt extraction, wood cutting and livestock breeding.

### Strategy of Vector Control implementation

A T0 basic entomological survey was conducted to orient of the implementation of vector control. This T0 survey was preceded by a geographical survey carried out in April 2017. This step allowed to identify and georeference several locations believed to be sites of human/tsetse contact (generally landing stages and mangrove channels). Based on this study and epidemiological data, 156 biconical traps [18] not impregnated with insecticide were set up across all of these sites to assess the presence of tsetse flies (Figs 1B and S1).

Following the T0 entomological survey, 31 of the 156 traps were selected as sentinel sites (one trap per site) for vector control monitoring. The selection of these 31 traps was based on three fundamental factors: epidemiological, ecological and entomological. The epidemiological factor was based on the number of g-HAT cases detected in the locality and on population dynamics. The ecological factor took into account the different biotopes favorable to tsetse and used by the populations to develop their activities. The entomological factor was based on the observed tsetse density or infection rate at each trap.

### Tiny Targets deployment

In Guinea, vector control against g-HAT is based on the deployments of deltamethrin impregnated tiny targets (Fig 1C) [10]. The first deployment of 5144 tiny targets was carried out from 26 December 2017–22 January 2018 (Fig 1A). According to the results of the T0 entomological survey, tiny targets were placed in places of human activity harboring the presence of tsetse flies (mangrove channels, landing stages, water points, rice fields, salt extraction sites, wood cutting, market gardening) in order to reduce human-tsetse contacts. The maximum distance between tiny targets on the mainland is 50 meters, while in mangrove channels it varies between 100 meters in narrow channels (less than or equal to 20 meters wide) and 200 meters in wide channels (over 20 meters wide).

The coordinates of each Tiny Target were recorded using the Map 64S GPS positioning system (Garmin), tracking and replacement in the field. In each village, one or two people (community agents) designated by the community assisted the central coordination team in carrying out this activity. In the field, installation was organized by teams of three people (one technician and two community agents). Logistics included 4x4 vehicles and motorbikes on the mainland and motorized boats on the islands. After the initial deployment tiny targets were replaced each year in 2019 and 2020.

All these activities were preceded by extensive community awareness campaigns involving film screenings about the disease, radio broadcasts, theatre performances and meetings to encourage participation.

### Entomological evaluations

**Fig 2:**
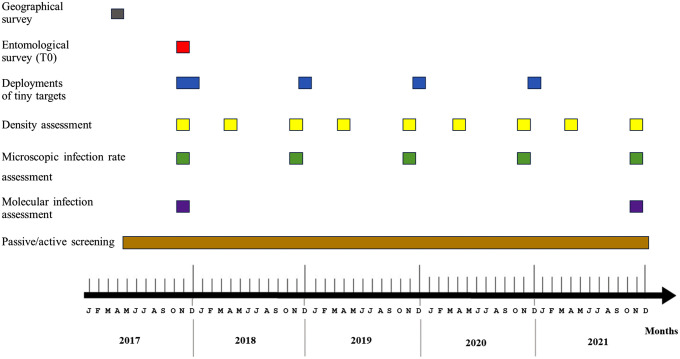
Flow chart showing the different activities implemented in the sleeping sickness focus of Forecariah through the study period 2017-2021.

**Fig 3:**
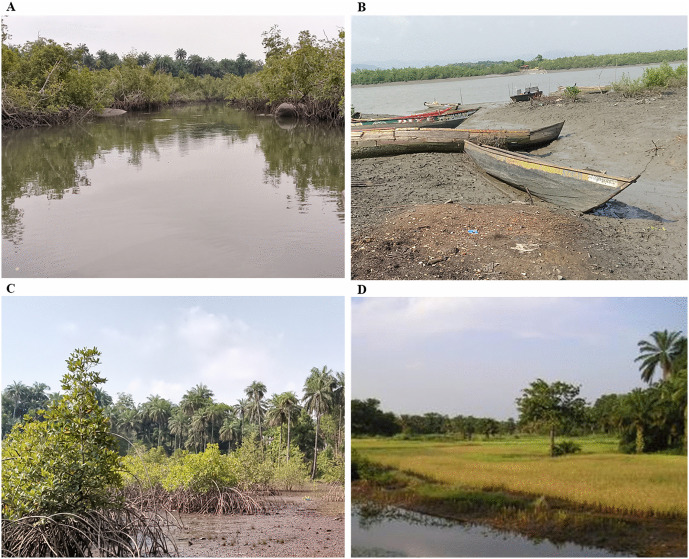
The characteristic biotopes of the Forecariah HAT focus: Channel (A); Landing stage (B); Mangrove continent interface (C); Rice field (D).

The overall strategy flow chart is shown in Fig 2. Every year, three and nine months after the annual deployment, the 31 biconical traps were installed at the sentinel sites (1 trap = 1 sentinel site), located in areas of human activity such as mangrove channels, landing stages, the interface between the mangrove and the mainland, rice fields (Fig 3) in order to assess the progress of the control program. Three main parameters were evaluated:

i. Tsetse fly density

First, to assess tsetse fly density, trapping was done at T0 then three and nine months after annual deployments. Density evaluation consists of assessing the density of tsetse through captures that were made over 48 consecutive hours with readings every 24 hours. For each trap, the apparent density (AD) is calculated as the average number of tsetse flies captured per day over two days and is reported as ‘flies per trap per day’ (F/T/D).

ii. Infection rate assessment by microscopy

Second, trypanosome infection rate was assessed by microscopy at T0 then once per year, 3 or 9 months after the deployment. During the midgut dissection process, all the fresh tsetse flies were dissected to facilitate the dissection and the microscopic reading. We expressed the infection rate by the number of traps with infected tsetse flies divided by the total number of traps.

iii. Infection rate assessment by molecular biology

Finally, to determine at T0 and T + 3years by molecular analysis the trypanosome infection rate, midguts were sampled after the dissection process. The strategy of sampling consisted, for each trap, to an exhaustive and individual sampling of the positive midguts and to a pooling to up to 10 midguts for the negative ones. Practically, to develop a cost-effective xenomonitoring system at these sites, the midguts of tsetse from the same traps that were free of parasites under the microscope were pooled in groups of 2–10 midguts in a 1.5 ml Eppendorf tube containing 70% ethanol. In addition, positive midguts (i.e., those in which trypanosomes were observed under the microscope) were placed in individual tubes containing 70% ethanol. In the field, these samples were stored at room temperature and then transported to the NTD-NCP molecular biology laboratory. Dissected and undissected tsetse flies were identified morphologically and then counted by trap.

### Molecular identification of parasite species

Molecular analysis of the collected tsetse midguts was carried out at the Program’s molecular biology laboratory based in Dubreka. DNA from gut pools and individual tsetse was extracted using a Qiagen kit [19]. A negative extraction control (water) was systematically added during each extraction run. Two successive final-point PCRs were used for the identification of the parasite species and the determination of the infection as described in the protocols below: TRYP1S/R used for the diagnosis of *T. brucei s.l., T. vivax, T. congolense savannah* and *forest* [20] and TgsGP [21] used to specifically identify *T. b. gambiense*. Indeed, TRYP1S/R PCRs were carried out on DNA extracted from the gut of captured flies (in pools and individually). Reference DNAs of each of the trypanosome species were systematically added during the PCRs in order to properly compare the banding pattern size. The samples showing amplifications at the size of 520 bp for *T. brucei sl* were selected to perform the TgsGP specific PCR. Positive TgsGP products were sent in France to Intertryp, the WHO Collaborative Center for African Trypanosomiasis diagnosis and sequenced by Sanger method.

### Medical monitoring

In Guinea, since 2016, medical surveillance has been based on both active mass screening campaigns and passive detection in 101 health posts and centres including 31 in Forecariah with three confirmation laboratories located in the country’s active foci (Boffa, Dubreka and Forecariah) [16]. For the passive screening process, registers indicating the symptoms of the disease for clinical suspicion have been developed and made available to the health agents who have been trained in these foci. When one of these signs is observed in an individual, the RDT g-HAT is performed immediately. If the RDT is positive, the individual becomes a serosuspected case and is referred for parasitological confirmation at one of the three centres. In parallel, active screening surveys are performed twice a year in each of the endemic focus. The algorithms used for these active screening campaigns has been recently presented in Camara et al [22].

For the purposes of this study, we extracted data from both types of screening (number of people tested and number of cases detected, as well as their geographical coordinates) based on the NTD-NCP database during the implementation period of the vector control intervention in Forecariah (2017–2025).

### Mapping

QGIS 3.16 was used to carry out the mapping. The GPS coordinates of the points recorded in the field in GPX format were projected (WGS 84) onto a base map of Google Earth and Open Street Map satellite imagery.

### Data collection, management and statistical analysis

All data (coordinates and biotopes) from tiny targets and traps at sentinel sites were collected on Kobotoolbox. These data were extracted in Excel format, the JMP software (*JMP 11,* SAS Institute Inc. 2013) and Prism 10 for Windows (version 10.6.1) were used for all statistical analyses and the graphic constructs.

i. T0 entomological survey

To compare tsetse catches (number of flies per trap per day) between traps set in different biotopes (mangroves or the mangrove/continent interface), we used Wilcoxon Kruskal–Wallis non-parametric tests, as neither the daily trap count (ADT) nor the log-transformed ADT were normally distributed. The proportion of traps with at least one infected fly in these different biotopes was analysed using the Fisher’s exact test.

ii. Entomological monitoring

The number of tsetse flies caught per trap per day from the 31 sentinel traps used to monitor tsetse densities in the vector control intervention area was analysed using general mixed-effects models (GLMMs) with the linear regression platform of JMP software. To assess the effect of different covariates, the monitoring period (late dry season in May or early dry season in November), the biotope in which the trap was placed (mangrove or mainland), and the presence or absence of Tiny Targets were specified as fixed effects, while the target location and sampling round were specified as random effects. Tsetse catches at each sampling round were compared to the numbers observed at T0 using a GLMM in which target locations within biotopes were specified as random effects and the sampling round as a fixed effect.

The percentage of flies with microscopic midgut infection at each time points was compared to the percentage observed at T0 by the Fisher Exact test.

A linear regression model was used to analyse the proportion of traps with positive microscopy and positivity to molecular tests, with the sampling round (T0 and T3 + 9M) and biotope specified as fixed effects and trap positivity weighted by the number of tsetse flies caught in the trap.

iii. Prevalence of HAT

The Pearson’s Chi-squared test with Yates’ continuity correction was used to compare the HAT prevalence observed in the focus (total number of active and passive HAT cases/ total number of people screened in active and passive screening) in 2017 and 2021.

## Results

### Strategy of Vector Control implementation

The data obtained through the entomological evaluation of the 156 traps enabled us to identify areas with a high density of tsetse flies (Table 1). The ADTs measured from traps in the mangrove biotope were significantly higher than those in the mainland (10.1 [6.6–13.6] F/D/T vs. 3.1 [1.9–4.2] F/D/T, respectively; p < 0.0001), and the flies were also significantly more infected (34.3% vs. 10.2% of positive traps; p < 0.0001). We therefore selected 31 sentinel sites based on these data, with 20 traps located in the mangroves and 11 on the mainland (S1 Fig), showing an ADT mean of 13.3 [7.1 - 19.5] F/D/T and an infection percentage per trap of 58.1%.

**Table 1:** Entomological data (AD and infected flies) assessed from the 156 biconical traps used during the T0 survey, according to the biotopes (mangrove vs mainland).

	Nb of traps	ADT [95% CI]	P-value*	Nb of traps with infected flies (%)	P-value**
**All traps T0**	156	6.1 [4.4 - 7.9]		33 (21.1)	
Biotope					
(i) mangrove	68	10.1 [6.6 - 13.6]		23 (34.3)	
(ii) mainland	88	3.1 [1.9 - 4.2]	<0.0001	9 (10.2)	<0.0001
**Sentinels Traps**	31	13.3 [7.1 - 19.5]		18 (58.1)	
Biotope					
(i) mangrove	20	17.2 [8 - 26.3]		14 (70)	
(ii) mainland	11	6.2 [1.5 - 11]	0.02	4 (36.4)	0.13

*Wilcoxon/Kruskal-Wallis non parametric tests.

**Fisher exact test.

The first VC campaign, performed in January 2018, involved deploying 5,144 Tiny Targets. The majority (75%) were deployed in the mangroves, while the remaining 25% were deployed on the mainland (e.g., landing stages, rice fields and the interface), in areas where local communities developed their activities (e.g., fishing, rice farming, collecting salt and cutting wood). The total coverage area in this focus was 638 km² corresponding to 8 tiny targets per km².

Throughout the four deployment campaigns (2018–2021), while the total number of Tiny Targets remained stable, their localization was slightly modified in order to adapt to the real-time entomological data obtained. These small modifications are depicted in S2 Fig.

### Impact of tiny target deployments on tsetse fly density

**Fig 4:**
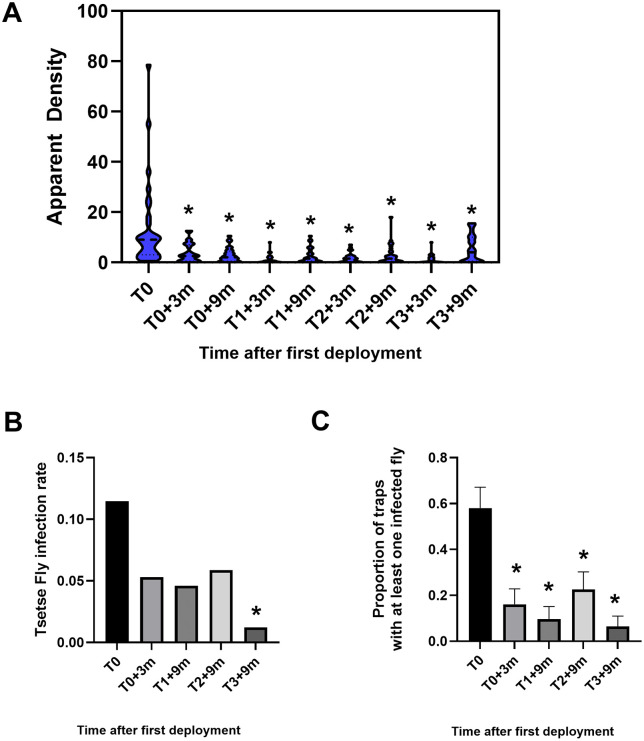
Evolution of (A) the tsetse fly apparent density (AD) through the 3 years of VC, (B) Tsetse fly infection rate and (C) the proportion of traps with at least one infected fly assessed by microscopy.

**Fig 5:**
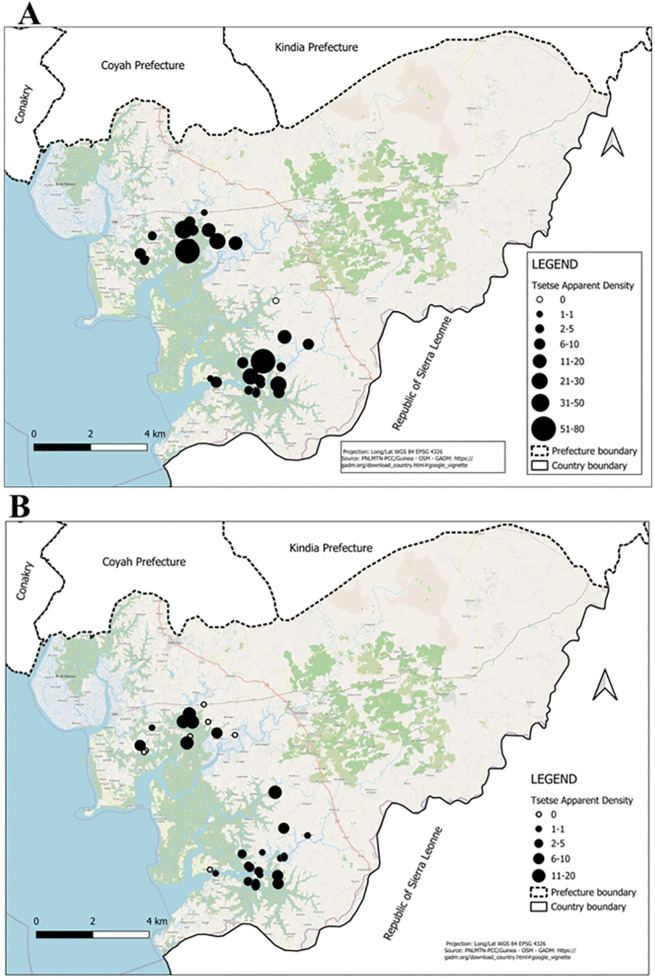
Distribution of tsetse densities before (A) and after 3 years (B) of vector control in the Forecariah HAT focus. This map was elaborated in-house with QGIS 3.28.12 from an OSM standard layer (www.openstreetmap.org).

After the deployment of the 5,144 tiny targets in the Forecariah focus in 2018, a fast and significant overall reduction in tsetse densities was observed with 825 tsetse caught in 2017 (mean ADT = 13.3F/D/T) compared to 67 (mean ADT = 1.1 F/D/T; p < 0.0001) after 18 months of VC (T1 + 3months), corresponding to a drastic reduction of 92% (Fig 4A). This high level of reduction was maintained at T2. After 3 years of VC, 336 tsetse flies were caught in 2021 (mean ADT = 5.4F/D/T; p = 0.003), still corresponding to a significant (p = 0.006) reduction rate of 59% as compared to T0 (Figs 4A and 5).

### Impact of vector control on the tsetse infection status

**Fig 6:**
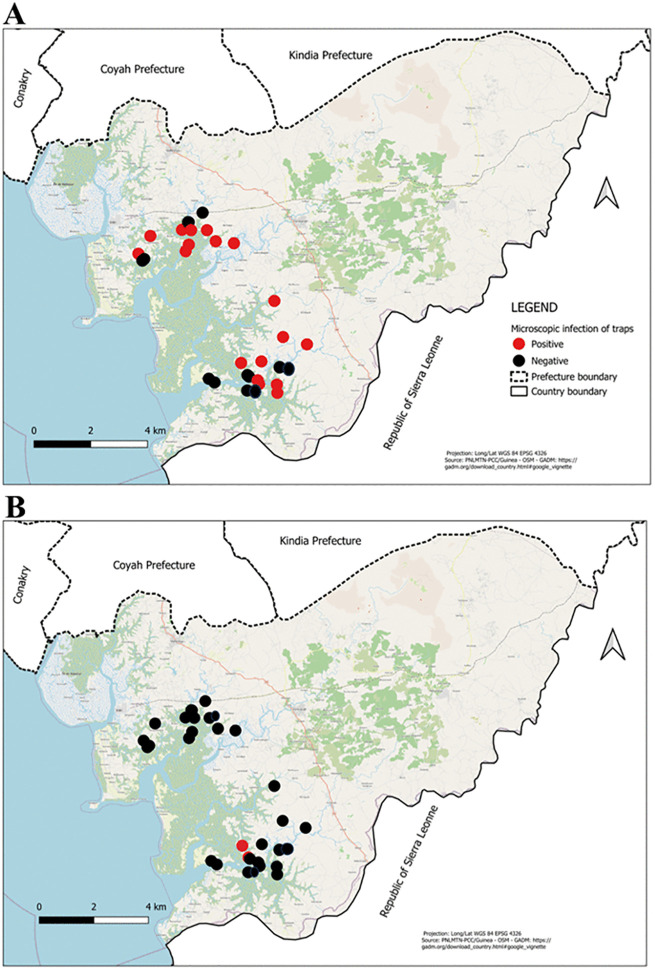
Distribution of microscopic infections found in tsetse before (A) and after 3 years (B) of vector control in the Forecariah focus. This map was elaborated in-house with QGIS 3.28.12 from an OSM standard layer (www.openstreetmap.org).

During the T0 entomological survey, microscopic examination of the midguts of 36/314 (11.5%) dissected tsetse flies revealed positive results. Throughout the three years of the VC measures, a progressive and significant decrease was observed, with only 2/164 (1.22%) infested tsetse flies found at the end of the survey (Fig 4B). The percentage of traps with at least one infected fly decreased significantly (p < 0.0001) from 58% (18/31) before VC measures were introduced, to 6% (2/31). This trend of continuous decrease was evident throughout the three years of VC measures (Figs 4C and 6).

The results of the linear regression model used to analyse the proportion of traps with positive microscopy and positivity to molecular tests, with the sampling round (T0 and T3 + 9M) are shown in the Table 2. The biotopes (mangrove vs mainland) were set up as fixed effects and trap positivity weighted by the number of tsetse flies caught in the trap. The decrease in infection rate was significantly (p < 0.001) greater in traps located in mangrove channels (70% to 5%) than in traps on the mainland (36% to 9%) (Table 2).

**Table 2:** Evolution of Trypanosome infection given per trap, assessed by microscopy and by molecular analysis before (2017) and after (2021) VC in the Forecariah HAT focus.

	Covariates	P-value	positive trap in 2017	positive trap in 2021
**Microscopy**	Monitoring survey	**<0.0001**	18 (58%)	2 (6%)
	Biotope	0.0001		
	Mangrove (20 traps)		14 (70%)	1 (5%)
	Mainland (11 traps)		4 (36%)	1 (9%)
**TRYP1SR all bands**	Monitoring survey	**<0.0001**	15 (48%)	8 (26%)
	Biotope	<0.0001		
	Mangrove (20 traps)		11 (55%)	5 (25%)
	Mainland (11 traps)		4 (36%)	3 (27%)
**TRYP1SR (520 bp - *T. brucei sl*)**	Monitoring survey	**<0.0001**	14 (45.2%)	7 (22.6%)
	Biotope	<0.0001		
	Mangrove (20 traps)		11 (55%)	4 (20%)
	Mainland (11 traps)		3 (27%)	3 (27%)
**TRYP1SR (750 bp - *T. congo*)**	Monitoring survey	0.34	2 (6.5%)	2 (6.5%)
	Biotope	0.02		
	Mangrove (20 traps)		1 (5%)	2 (10%)
	Mainland (11 traps)		1 (9%)	0
**TRYP1SR (310 bp - *T. vivax)***	Monitoring survey	**<0.0001**	2 (6.5%)	5 (16.1%)
	Biotope	0.46		
	Mangrove (20 traps)		1 (5%)	3 (15%)
	Mainland (11 traps)		1 (9%)	2 (18%)
**TgsGP (308 bp - *T. brucei gambiense)***	Monitoring survey	0.99	0	2 (3.2%)
	Biotope	0.99		
	Mangrove (20 traps)		0	1 (5%)
	Mainland (11 traps)		0	0

NB: Nominal logistic regression models for Trap positivity. Trap positivity rates were weighted by the number of tsetse fly captured in the trap.

Molecular analysis (Table 2) at T0 showed that *T. brucei. sl* was the most prevalent trypanosome species with 45.2% of sentinel traps showing positivity to the 520 bp band. *T. congolense* (750 bp) and *T. vivax* (310 bp) were also identified in 6.5% of sentinel traps during the T0 entomological survey. After three years of vector control, a significant decrease of the PCR positivity was observed for *T. brucei sl* (45.2% versus 22.6%; p < 0.001), especially in the mangrove areas. The prevalence of positivity remained stable for *T. congolense* and a slight by significant (p < 0.0001) increase was observed for *T. vivax* (6.5% versus 16.1%), independently of the biotope. Only one midgut pool collected in 2021 tested positive and the sequence of the amplicon confirmed that the PCR product was *T. brucei gambiense* (100% homology with the Gene Bank accession number FN555988 reference isolate).

### Evolution of the HAT prevalence in the Forecariah Focus

**Fig 7:**
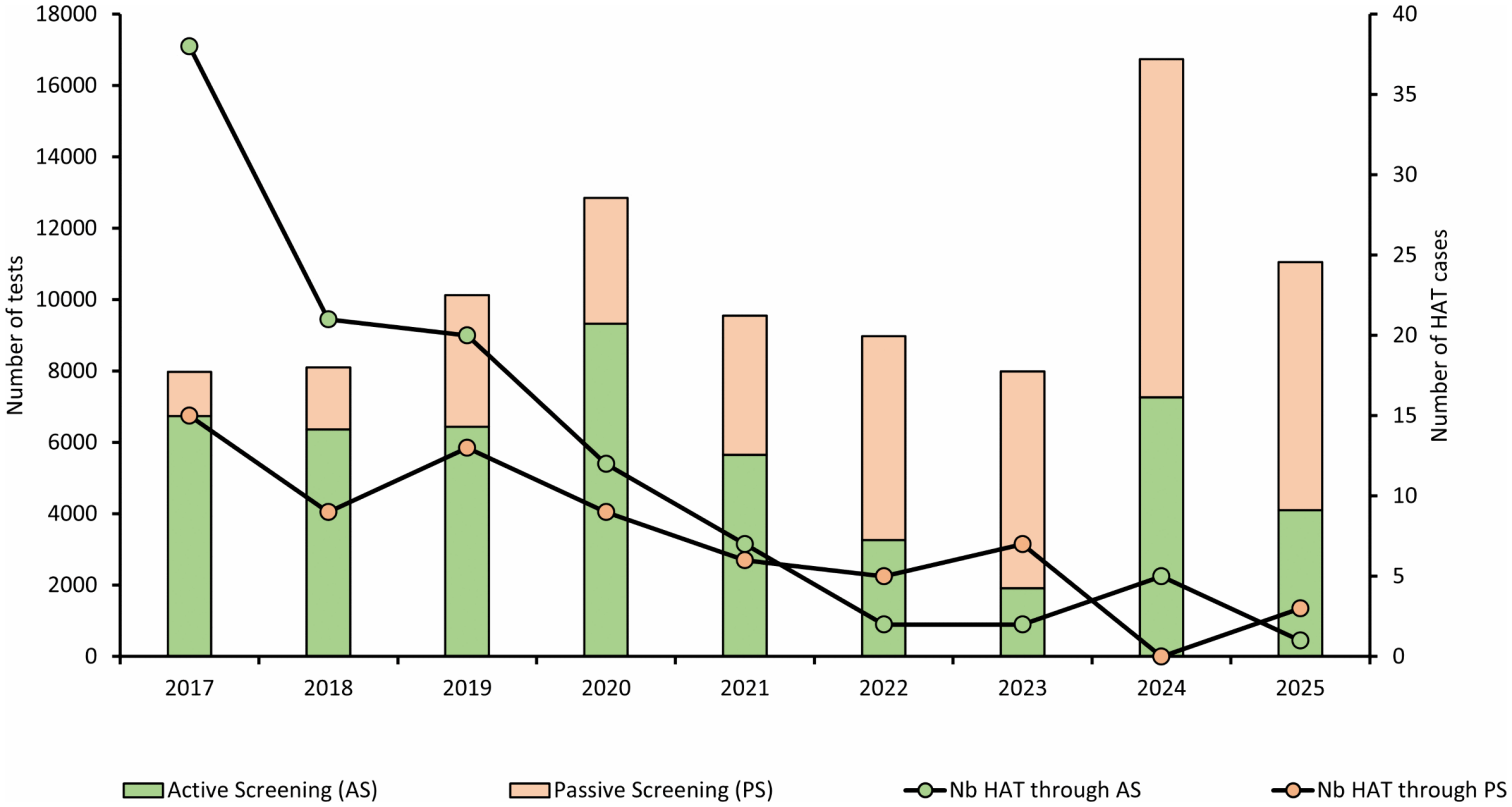
Evolution of the number of HAT cases detected through passive and active screening methods in Forecariah from 2017 (start of vector control) to 2025. (www.openstreetmap.org).

**Fig 8:**
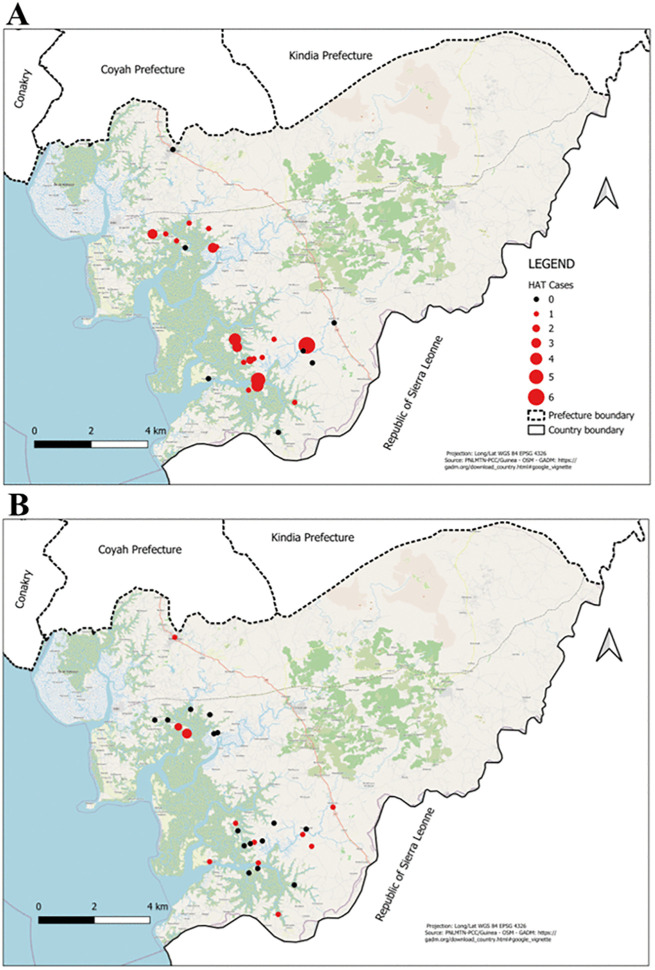
Distribution of cases HAT before (A) and after 3 (B) of vector control in the Forecariah focus. This map was elaborated in-house with QGIS 3.28.12 from an OSM standard layer (www.openstreetmap.org).

Before VC, a total of 53 patients were identified (including 38 and 15 in active and passive screening respectively) out of a population of 8070 screened in the Forecariah focus in 2017 (Fig 7). After only one year of VC implementation, cases detection dropped to 30 (21 by AS and 9 by PS). Four years after the start of the intervention, only 13 out of 9560 people screened were confirmed as g-HAT patients (6 by AS and 7 by PS), representing a 75% significant decrease of the overall disease prevalence from 0.66 to 0.14% (p < 0.0001)**.** The geographic distribution of cases HAT before (2017) and after (2021) VC is shown in Fig 8. Given these encouraging results, VC was maintained. The significant downward trend continued in the years 2022–2025, with a prevalence of 0.04% in 2025 corresponding to a 94% decrease compared to 2017 (Fig 7).

## Discussion

Tiny target deployments is a frequent strategy used in combination with medical screening in g-HAT control programs [10,13]. These combined strategies were shown to accelerate the elimination process in several active foci in Uganda, Côte d’Ivoire, Chad [7] Cameroon [23], Democratic Republic of the Congo [12]. In Guinea, this strategy was first implemented in Boffa focus in 2012, then spread to Dubreka and Forecariah in 2016 and 2018 respectively [8].

In g-HAT foci where tsetse flies have a riverine habitat restricted to the hydrographic network such as Chad and Uganda, linear tiny target deployment are very efficient, leading to a drastic reduction of tsetse densities (generally over 95%) [11]. The situation is very different in biotopes such as mangroves in Guinea, where tsetse are much more largely distributed with very large population sizes [11] and in which large areas are totally inaccessible to any tsetse control tools. In Guinea, where all active foci are located in mangrove areas along the coast, the strategy adopted by the NTD-NCP to fight against this disease consists in controlling tsetse fly populations in order to reduce contacts between humans and vectors which tends to interrupt the trypanosomes transmission [24]. It therefore targets areas shared by humans and tsetse flies that are conducive to transmission. The strategy combining VC with medical activities was first tested in Boffa in 2012 in a two-arm trial, in which tiny target deployments were initiated in the eastern part of the focus area only, in addition to medical screening. This showed that, in addition to medical screening and treatment activities, tiny target deployments enabled the elimination process to be significantly speeded up [10]. This is further confirmed in this study led in Forecariah, where the prevalence of the disease decreased from 0.66 to 0.14% four years after the introduction of tiny target deployments. This impact appears sustainable, as the decrease has been maintained through 2025, year in which prevalence fell to 0.04%. The strong impact of introducing vector control alongside HAT screening strategies is further supported by a transmission model fitted to historic Guinean data. This model shows a sharp decrease in estimated number of new infections in 2018, just one year after VC was introduced to the area [25].

Beyond their impact on disease prevalence/incidence and vector densities, the effects of control strategies on the life cycle of tsetse-transmitted trypanosomes had been poorly investigated in Guinea. In this study, in addition to describing the impact of vector control on tsetse flies populations in the Forecariah district and the evolution of disease prevalence, we document the impact of vector control measures on the circulation of trypanosome species within the focus.

As the VC in Guinea covered all the 3 active foci, no control areas, without Tiny Target deployments, and with similar eco-epidemiological features, were selected. Although this can be considered a limitation of our study, the rapid and significant decrease in g-HAT prevalence observed in humans in Boffa [10] encouraged the NTD-NCP to efficiently protect the largest at-risk population in the remaining foci as quickly as possible.

As commonly observed during VC campaigns against tsetse flies [26], a first step of drastic decrease of ADT (known as suppression step) was observed 18 months only after the first deployment. The most marked reduction rates were observed in mangrove biotopes harboring the highest densities of tsetse flies. This is particularly important as these sites represent epidemiological high-risk spots for *T. b. gambiense* transmission. In mangrove areas, which are complex and often inaccessible, channels and landing stages are the main routes used by local populations for various activities (fishing, rice farming, wood cutting and salt extraction) [27]. In these channels, the highest densities of tsetse flies are recorded. The abundance of flies in this area could be linked with a favorable hunting ground for their blood hosts, particularly crocodiles, monitor lizards and humans, who can move around easily there [28]. In contrast, no significant impact was observed in mainland sites where tsetse densities where already lower before the first deployment of tiny targets. In such biotopes, increasing the number of tiny targets or using the “cross” tiny target that was recently shown to be more attractive and more resilient to climatic disturbances [29] could represent a solution to improve vector control in these areas.

Even though the total number of deployed Tiny Targets remained constant over three years, their distribution changed slightly. For example, in Forodougou, where ADTs were low (ranging from 0 to 2.5 F/D/T during the first seven evaluation surveys, with no infected flies), 35 Tiny Targets were removed in 2021 (shown in red in S2 Fig). Consequently, an increase in the ADT (15.5 F/D/T) was observed, which was not only caused by the absence of VC, but also by anthropogenic modifications such as small-scale deforestation that could have disturbed the ecology of the tsetse flies. The same has been observed for four other sentinel traps in Gbaran and Tady Kalé Koléah (S2 Fig), which could explain the slight increase in ADT during the last evaluation survey.

Together with the impact of tiny targets deployments on tsetse fly densities, a major decrease of the prevalence of microscopically positive trypanosome midgut infections was observed in tsetse (from 10.8% to 1.2%). The difference was highly significant in mangrove sites whereas no significant differences were observed in mainland sites where fewer impact of the intervention was observed on tsetse densities. A similar decrease has been recently reported in Cameroon by Tanekou et al with a 75% infection rated drop from 21.2% to 5.06% through 2 years of VC [23].

According to the TRYP1S/R PCR, *T. brucei sl* was the most frequently detected species with 45.2% of traps showing positivity to the 520pb band in 2018 and was also the most impacted with only 22.6% of trap positive in 2021 (p = 0.05). The percentage of traps positive to *T. congolense* and *T. vivax* remained stable over the study period. The percentage of traps positive by PCR was higher than by microscopy illustrating the higher sensitivity of the PCR to detect parasite DNA [30]. Nevertheless, it is also likely that PCR positive but microscopically negative guts did not contain multiplying procyclic forms but rather DNA from blood forms acquired during recent blood meals. This is likely the case for *T. vivax*, from the *Duttonella* subgenus known to be restricted to the proboscis [31]. It could also possibly be the case for the TgsGP positive PCR (negative by microscopy) that could be the result of a recent blood meal taken by the tsetse fly on a g-HAT patient. As proboscis and salivary glands where not collected during these surveys, it was not possible to assess the impact of the vector control intervention on the proportion of flies carrying mature infections. Nevertheless, it shows a major impact on the circulation of trypanosomes from the *Trypanozoon* subspecies (to which *T. b. gambiense* belongs) in the vector control area.

The percentage of traps testing positive for *T. congolense* remained stable throughout the study, whereas the percentage testing positive for *T. vivax* increased significantly. One possible explanation for these two observations could be that domestic animals, in particular cattle, are the main mammalian hosts of these two trypanosome species in this area. As domestic animals are mainly present in mainland areas, the poor efficacy of tiny target deployments in these areas could explain why no effect was observed for these trypanosome species. Alternatively, the study may have had insufficient power to detect small effects, as the prevalence of *T. congolense* in the tsetse fly gut was low prior to the vector control.

Unlike *T. vivax* (*Dutonnella*) and *T. congolense* (*Nannomonas*), for which the infective forms are found in the proboscis, the transmission of *Trypanozoon* species requires infection of the tsetse salivary glands [30]. Since only a small percentage of *T. brucei* infections are becoming mature, this particularity of the life-cycle may partly explain why transmission levels for this species were drastically reduced, despite the moderate effects of the vector control campaign on tsetse densities.

Examination of fresh dissected flies in the field is fastidious, time consuming and requires important technical skills limiting its application in the field at large scales. Here we show that pooling the tsetse guts or possibly entire flies caught from single traps with subsequent PCR analysis (TRYP1S/R and TgsGP on TRYP1S/R positive) can represent a powerful and cost-effective system applicable at much larger scales to monitor the population of trypanosomes circulating in a focus. Although studies conducted on animal reservoirs in Boffa in 2012 [27] did not detect the presence of any of these trypanosomes in domestic animals, recent studies conducted in ‘Guinée Forestière’, a former focus of HAT in Guinea [32] and in Cameroon, have shown the presence of *T. b. gambiense* in domestic and wild mammals [33–35]. In such a context where fauna could play the role of reservoirs, the xeno-monitoring systems are likely to become increasingly useful especially in foci where human cases are becoming rare and where vector control measures are scaled down in order to detect a potential re-emergence of *T. b. gambiense* transmission in a timely manner.

## Conclusion

Like in Boffa, a significant decrease of the human disease prevalence has been observed in the Forecariah focus shortly after the introduction of tiny target deployments. This happened despite a relatively modest effect on tsetse densities as compared to other vector control campaigns where tsetse can be reduced to very low levels. Here we show that in addition of reducing human tsetse contacts in high-risk spaces, the tsetse control strategy also impacted drastically the probability of finding a *Trypanozoon* infected tsetse, thus synergizing the impact on transmission.

## Supporting information

S1 Fig Distribution of the 156 traps used during the T0 survey. This map was elaborated in-house with QGIS 3.28.12 from an OSM standard layer (www.openstreetmap.org).
(TIF)

S2 Fig Evolution of the distribution of the Tiny Targets through the 4 deployments. This map was elaborated in-house with QGIS 3.28.12 from an OSM standard layer (www.openstreetmap.org).
(TIF)

S1 File Entomological database. (XLSX)

S2 File Database tiny target. (XLSX)

S3 File Medical database. (XLSX)
