# Supplementary figures and images for: Xeno-monitoring the impact of Vector Control on trypanosome transmission in the Forecariah sleeping sickness focus (Guinea)
Source: PLoS Negl Trop Dis. 2026 Jun 5;20(6):e0013598. doi: 10.1371/journal.pntd.0013598 (PMC13258148; doi:10.1371/journal.pntd.0013598)

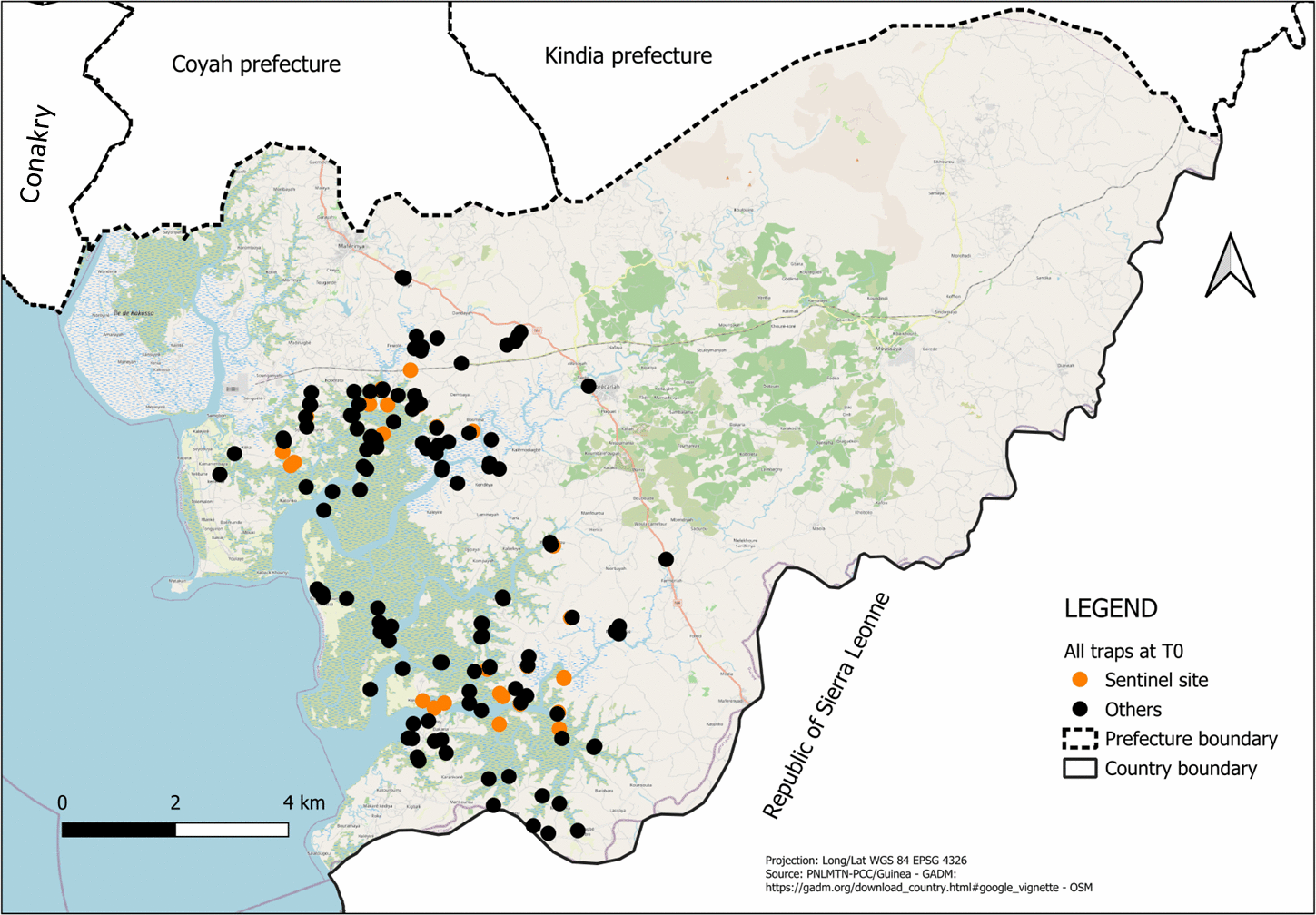

Supplement: S1 Fig — This map was elaborated in-house with QGIS 3.28.12 from an OSM standard layer (www.openstreetmap.org). (TIF) [file pntd.0013598.s001.tif]

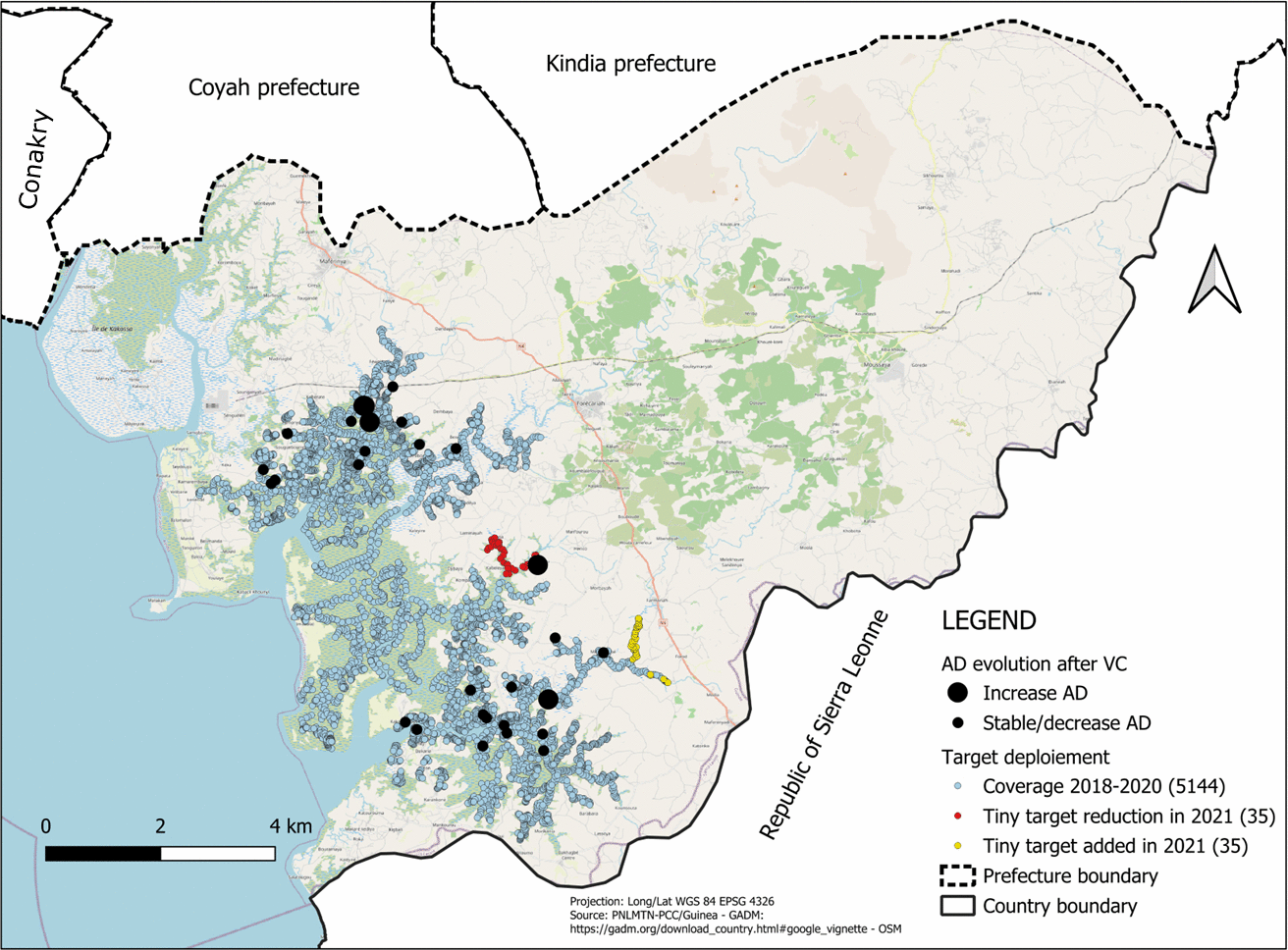

Supplement: S2 Fig — This map was elaborated in-house with QGIS 3.28.12 from an OSM standard layer (www.openstreetmap.org). (TIF) [file pntd.0013598.s002.tif]
